# Brain health assessment. An exploratory review of tools related to its cognitive dimension

**DOI:** 10.1016/j.cccb.2023.100188

**Published:** 2023-10-02

**Authors:** Alessia Nicotra, Giorgia Maestri, Emilia Salvadori, Leonardo Pantoni

**Affiliations:** a Neurology Unit, Luigi Sacco University Hospital, Milan, Italy; b Neuroscience Research Center, Department of Biomedical and Clinical Sciences, University of Milan, Milan, Italy

**Keywords:** Brain health, Assessment, Screening tools, Cognition, Neuropsychology, Out-patients services

## Abstract

**Background:**

Brain health is an evolving concept and relates to physical and mental health, social well-being, productivity, creativity. Brain health has several dimensions (cognitive, motor, functional, social, and emotional), and should be recognized as one top global priorities of health policies. The purpose of this paper is to provide a summary of tools developed for assessing the cognitive dimension of brain health in the out-patient services.

**Methods:**

A literature search on PubMed was performed (from inception to May 31, 2023). We identified cognitive tests, functional and psychological scales, and focused on screening tools specifically proposed to characterize cognition within the construct of brain health, comparing them with common global screening tests.

**Results:**

Among 1947 records, we identified 17 cognitive screening tools used in the context of brain health assessment, of which four were ad hoc developed: Brain Health Assessment (BHA), Brain Health Test (BHT), Brain Health Test-7 (BHT-7), and The Cogniciti Brain Health Assessment. The four tests have administration time ranging from 4 to 30 min, and different administration methods (paper-and-pencil or tablet-based). All four tools assess memory and other cognitive domains. Specific cut-offs have been identified for BHT and BHT-7, while the other tools have automated scoring systems. All but one test also assess other dimensions. Compared to commonly used cognitive screening tests, the brain health tools are less widely used, translated, and validated.

**Conclusions:**

The concept of brain health is new and requires further validation of tools for its assessment, especially for the cognition dimension.

## Highlights

• Brain health is a relatively new concept that encompasses many dimensions.
• Ad hoc screening tools have been developed to measure brain health.
• Compared to usual cognitive screening tools brain health tests are multidimensional.
• Until now, brain health tools are scarcely used, translated, and validated.

## 1 Introduction

Brain health is an evolving concept, increasingly used because of the worldwide aging of population, and promoted by clinical and academic neuroscience centers, seen in news journals, and endorsed by the general public. Despite the diffusion and utilization of the term, a clear, coherent, and comprehensive definition of brain health is still lacking [1].

A position paper developed by the World Health Organization (WHO) provides a conceptual framework of brain health and brain health optimization, defining it as the “*state of brain functioning across cognitive, sensory, social-emotional, behavioral, and motor domains, allowing a person to realize their full potential over the life course. Continuous interactions between biological determinants and a person's environment lead to lifelong adaptation of brain structure and functioning. Optimizing brain health improves mental and physical health and creates positive social and economic impacts, all of which contribute to greater well-being and help advance society, irrespective of the presence or absence of disorders*”.

According to this definition, brain health appears to be a multidimensional construct and involves several domains of brain functions such as cognition, emotions, and motor functions [1]. In this regard, brain health represents a state characterized by the correct functioning of different cerebral processes such as memory, language, executive functioning, mood, etc. These domains are largely attributable to the functions of the brain and can be operationally defined and measured; are influenced by environment, behaviors, and disease; and are potentially modifiable if changes are detected early enough [2]. Brain health may therefore be defined as the preservation of optimal brain integrity and mental and cognitive function at a given age in the absence of overt brain diseases that affect normal brain function [3].

However, brain health is a dynamic state along the life course; in this sense, brain health may change over time, becoming better or worse, depending on the presence and modification of risk and protective factors, in the context of aging-related changes.

Overall, there is a need for a broad definition of brain health that goes beyond the mere absence of structural and functional diseases and encompasses the potential assessment and monitoring of individuals. Optimal brain health may theoretically be defined as an optimal capacity to function adaptively in the environment [2].

Brain health should be recognized as a global public health priority, and it is therefore necessary to develop measures and definitions to enhance research and policy and implement strategies of prevention to support and maintain brain health [4].

Given the multidimensionality of the construct, collecting the full spectrum of brain health across all domains of brain functioning and accounting for all determinant variables is demanding. So, operationalizing brain health and developing validated tools and scales to measure brain health will be critical.

An ideal brain health assessment should be performed at the primary care level where it would efficiently detect all types of early decline, would simultaneously provide valid scores of key cognitive domains and level of functional impairment, and could be used by health providers, along with other clinical information, to evaluate if a patient meets diagnostic criteria for example for common cognitive syndromes [5].

However, currently there is no simple, direct, or comprehensive measure of brain health across the life course [6]. Inexpensive, easily translatable, and requiring no specialist equipment tools are the best candidates for brain health assessment [1].

Starting from a brief revision of tools for the office-based assessment of brain health, this explorative review focused on those with assessment of its cognitive dimension. Specifically, we aimed at exploring the structure of screening tools created ad hoc for brain health, and at comparing them with other cognitive screening instruments used in clinical practice.

## 2 Methods

A literature search in PubMed database was performed from inception to May 31 2023, using the following keywords in the title or abstract: “brain health” OR “brain-health” AND “battery” OR “screening” OR “questionnaire” OR “cognition” OR “assessment” OR “evaluation” OR “test” OR “scale” OR “tool”. No restriction of language or year of publication was adopted. Two researchers independently conducted the study selection and data extraction. The results were exported to Rayyan (https://www.rayyan.ai/), and subsequently, duplicates were removed. The titles and abstracts of the identified studies were screened and then the full texts of relevant studies were searched and further screened to characterize tools used to identify brain health (both in cognitive and psychological dimension). Inclusion criteria included papers describing original studies on the topic of brain health citing the specific tools used to assess it. We considered cognitive screening tests, neuropsychological battery, and scales assessing independence in daily living, mood, quality of life and other psychological features, focusing then on cognitive screening tests specifically developed to investigate the construct of brain health. Exclusion criteria were single case reports and reporting only non-psychological or non-cognitive instruments (such as gait scales).

## 3 Results

An initial screen on the PubMed database returned 1947 records. Through the abstract and full-text screening of these articles, 17 cognitive screening tools used in the context of brain health assessment were identified. In addition, we identified six neuropsychological test batteries, four multicomponent tools, four scales assessing quality of life, eight mood scales (including depression, anxiety, and stress), seven scales assessing independency in daily life, and 12 additional scales assessing other features such as quality of sleeping, subjective cognitive decline, and healthy aging activity engagement (Table 1).

**Table 1:** Tools that assess different dimensions of brain health.

Cognitive dimensions	Tools
Cognitive screening tests for brain health	• Brain Health Assessment (BHA) • Brain Health Test (BHT) • Brain Health Test-7 (BHT-7) • The Cogniciti Brain Health Assessment
Widespread cognitive screening tests	• Addenbrooke's Cognitive Examination (ACE) • Alzheimer's Disease Assessment Scale-Cognitive Subscale (ADAS-Cog) • Brief Test of Adult Cognition by Telephone (BTACT) • Clinical Dementia Rating Scale (CDR) • Dementia Screening Interview (AD8) • Mattis Dementia Rating Scale (MDRS) • Memtrax computerized memory test • Mini-Cog • Mini Mental State Examination (MMSE) • Montreal Cognitive Assessment (MoCA) • Short Portable Mental Status Questionnaire (SPMSQ) • Telephone Interview for Cognitive Status (TICS) • The seven minutes screen (7MS)
Neuropsychological battery	• Cambridge Neuropsychological Test Automated Battery (CANTAB) • CogState Brief Battery (CBB) • Consortium to Establish a Registry for Alzheimer's Disease (CERAD) • NeuroCognitive Performance Test (NCPT) • Repeatable Battery for the Assessment of Neuropsychological Status (RBANS) • Rivermead Behavioural Memory Test (RBMT)

Other psychological dimensions	Tools
Multicomponent tools	• General Health Questionnaire (GHQ) • Healthy Aging Instrument (HAI) • National of Institute of Health Toolbox (NIH) • 36-item Short-Form Survey (SF-36)
Functional/activity status	• Activity of Daily Living (ADL) • Cognitive Activity Scale (CAS) • Cognitive and Leisure Activity Scale (CLAS) • Everyday Cognition (Ecog) • Florida Cognitive Activity Scale (FCAS) • Functional Activities Questionnaire (FAQ) • Instrumental Activity of Daily Living (IADL)
Mood scales	• Apathy Evaluation Scale (AES) • Depression Anxiety Stress Scales (DASS-21) • Geriatric Depression Scale (GDS) • Personal Health Questionnaire Depression Scale (PHQ-8) • General Anxiety Disorder-7 (GAD-7) • Self-Rating Anxiety Scale (SAS) • Oxford Happiness Questionnaire (OHQ) • Perceived Stress Scale (PSS)
Quality of life scales	• Neurological Quality of Life (NeuroQoL) • Quality of life scale (QOLS) • Satisfaction With Life Scale (SWLS)
Others	• Beijing Aging Brain Rejuvenation Initiative - Subjective Cognitive Decline (BABRI-SCE) • Subjective Cognitive Decline Questionnaire (SCD-Q) • Perceived Deficit Questionnaire (PDQ) • Brain Health Self-Efficacy Scale (BHSES) • Connor-Davidson Resilience Scale (CD-RISC) • Engagement in Meaningful Activities Survey (EMAS) • Global Physical Activity Questionnaire (GPAQ) • Healthy Aging Activity Engagement scale (HAAE) • Epworth Sleepiness Scale (ESS) • Pittsburgh sleep quality index (PSQI) • Self-Care Chronic Illness Addresses Inventory (SC-CII) • Social Support Questionnaire (SSQ)

### 3.1 Cognitive screening tests specifically developed for the construct of brain health

Of the 17 cognitive screening tests identified, four were ad hoc developed to measure brain health. In the following sections, the structures and contents of brain health tools will be described and compared with screening instruments widely employed in clinical settings (Montreal Cognitive Assessment, MoCA; Mini Mental State Examination, MMSE).

#### 3.1.1 Brain health assessment (BHA)

The Brain Health Assessment (BHA) is a 10-min, tablet-based, brief cognitive screening.

The tool includes four cognitive subtests (memory, executive functions and speed, visuospatial skills, and language) and the Brain Health Survey (BHS) that comprises twelve questions from Everyday Cognition scale (ECog-12) [7] and additional nine questions. The survey is addressed to an informant and is self-administered to evaluate change in patients’ functional level and emergence of new neurocognitive symptoms over the past 5 years. It also features an automated text-based report that summarizes the performance and supports care decisions.

The BHA provides valid subscores of domains of cognition and function that are important for differential diagnosis and demonstrates excellent combined sensitivity and specificity to detect dementia and mild cognitive impairment (MCI) due to diverse etiologies, in the primary care setting. There is no global score or cut-off. The authors emphasize that the use of a single global score entail the risk of not detecting specific deficits [5]. Additionally, BHA was designed to minimize language, ethnicity, and education-related biases in the detection of cognitive impairment.

BHA is validated for use in research and clinical care. Its brevity, automated scoring, sensitivity, specificity, and the minimal training requirements for clinical staff are attributes that may contribute to its adoption in the primary care setting.

#### 3.1.2 Brain health test (BHT)

The Brain Heath Test (BHT) is a 4-min cognitive screening test. It was developed in 2015 by the Taiwan Dementia Society whose purpose was to develop an easy-to-use clinical tool to both identify dementia patients based on risk factors and perform a focused cognitive screening test [8]. The tool includes an analysis of the risk factors and a brief cognitive test (BHT-cog).

The risk score (RS) is calculated relying on information provided by the patient or the caregiver, and it includes age, education, sex and comorbidities. The final score ranges from 0 to 18. Authors defined patients as high-risk of dementia when they met one of the following criteria: 1) subjective memory decline regardless of whether it is based on patient's or informants’ report, 2) need of help from others to manage money or medications, or 3) total RS ≥8.

The BHT-cog consists of assessments for orientation to time, immediate and delayed verbal memory recall, and verbal fluency, with a total score ranging from 0 to 16 (clock drawing task was excluded because it was not completed by more than one-third of the participants, including many healthy subjects). A cut-off of 10 was identified at the BHT-cog to detect dementia patients. The degree of education did not affect the cut-off value.

A multicenter study using this test on healthy subjects, MCI and dementia patients was conducted including 813 subjects with more than 49 years. A high sensitivity (98.1 %), but a low specificity (44 %) was found for the identification of subjects classified at high-risk according to RS. A high sensitivity (91.5 %) and specificity (87.3 %) were found for BHT-cog to discriminate between healthy subjects and dementia patients. If the RS and BHT-cog were combined, the sensitivity was 90.8 %, and the specificity was 92.2 %.

However, the BHT-cog only showed a limited specificity (64.9 %) in discriminating between MCI and dementia patients when the cut-off value is set on 10. In addition, to the best of our knowledge, the tool has only been developed, validated, and used in the Taiwanese population.

#### 3.1.3 Brain health test-7 (BHT-7)

The brain health test-7 (BHT-7) is a simple cognitive screening tool developed in Taiwan to help primary care physicians identifying patients with MCI and early dementia among patients with memory complaints or at risk for dementia.

The BHT-7 is based on the original Brain Health Test (BHT) cognitive test proposed by Tsai and colleagues with the addition of visuospatial construction and frontal lobe function tests. The test takes 5–7 min to administer.

The cognitive test includes the orientation to time, immediate and delayed recall of five items, categorical verbal fluency test (listing four-legged animals in 1 min), visuospatial construction (cube drawing and clock drawing), and frontal lobe function test (Luria's hand test). The total score of BHT-7 ranges from 0 to 23.

In comparison with BHT, the BHT-7 showed better sensitivity in differentiating MCI from the normal group, with a cutoff value of 17/18 (sensitivity = 86 %, specificity = 76 %, area under curve = 88 %). However, it was only validated on the Taiwanese population. It has the limitation of being validated on a small sample size, and it was not designed to differentiate different subtypes of dementia. Thus, future studies with a larger sample size seem needed to further validate the results and test their generalizability.

#### 3.1.4 The Cogniciti brain health assessment

The Cogniciti Brain Health Assessment is a free online cognitive test that takes between 20 and 30 min to complete (https://cogniciti.com/Test-Your-Brain-Health/Brain-Health-Assessment) [9]. It does not require the presence of an examiner to be performed.

Authors selected four tasks of memory and executive attention based on the evidence that such abilities decline in normal cognitive aging [10]. The cognitive test includes: a spatial working memory task, where subjects are required to correctly locate some pairs of hidden shapes in a 4 × 3 matrix; a Stroop task; a face-name association task; a variation of Trail making Test part B (TMT-B). At the end of the TMT-B, the subject is asked to remember again the position of the shapes shown in the first task. Four parallel versions are available.

When accessing the online assessment, some demographic information is requested, such as age, schooling, native language, ethnicity. Self-assessment questions are asked pertaining to any changes in cognition or in daily autonomy.

Troyer et al. calculated normative data in a sample of 396 healthy subjects and transformed the performance into z scores. The performance turned out to be influenced by age, education and the version of the test used.

The Cogniciti Brain Health Assessment demonstrated adequate internal consistency, test-retest reliability, alternate version reliability, construct validity and adequate convergent validity. This test showed an overall accuracy in classifying MCI comparable to Montreal Cognitive Assessment (MoCA), with some advantages compared to MoCA in the screening of higher functioning people [11,12]. Its availability as a free online tool makes it easy to collect data on large samples [13].

The test was developed in the United States of America and is currently available only in English.

Table 2 summarizes the main characteristics of the four tests as compared with two largely used global cognition screening tests (MoCA and MMSE).

**Table 2:** Comparison of the main features of two widely used cognitive screening tools (MoCA, MMSE) and tools specifically related to brain health (cognitive dimension). Cognitive domains defined according to the original papers.

Tools	Orientation	Memory	Working memory	Attention	Language	Visuospatial	Executive functions	n subtest	Other brain health dimensions evaluated	Time of execution	Paper-and-pencil/tablet-based	Translations in different languages	Global score and cut-off
MoCA	X	X		X	X	X	X	12	No	10–15 min	Paper-and-pencil and tablet-based	Yes (*n* > 10)	Yes (range 0–30)
MMSE	X	X		X	X	X		10	No	10 min	Paper-and-pencil	Yes (*n* > 10)	Yes (range 0–30)
BHA		X			X	X	X	4	Yes (BHS for daily independence)	10 min	Tablet-based	Yes (*n* = 3)	No
Cognicity brain health assessment		X	X	X			X	4	Yes (Biographical informations; subjective cognitive impairment; daily independence)	20–30 min	Tablet-based	No	No
BHT	X	X				X		4	Yes (risk factors)	4 min	Paper-and-pencil	No	Yes (range 0–16)
BHT-7	X	X			X	X	X	7	No	5–7 min	Paper-and-pencil	No	Yes (range 0–23)

*List of abbreviations:* BHA, brain health assessment; BHS, brain health survey; BHT, brain health Test; BHT-7, brain health test-7; MMSE, mini mental state examination; MoCA, Montreal cognitive assessment.

### 3.2 Analysis of the tools proposed for brain health assessment

The analysis of the four tests related to the construct of brain health shows administration times ranging from 4 min for the BHT to 30 min for the Cogniciti Brain Health Assessment. The methods of assessment are either pencil and paper or tablet-based.

All the four tools assess multiple cognitive domains, among which memory is always considered. Also, executive functions are evaluated by all instruments except the BHT, while the assessment of visuo-spatial functions is disregarded in Cogniciti Brain Health Assessment alike language. The two tablet-administered instruments (BHA and Cogniciti Brain Health Assessment), unlike the other tools, do not present an overall score and relative cut-offs; the scores are instead automatically converted into z scores. In BHA, the software automatically provides subscores obtained for the different cognitive domains investigated, while the Cogniciti Brain Health Assessment software automatically provides the percentile in which the subject's overall performance lies, without providing specific scores.

An important aspect concerns the non-cognitive brain health dimensions that are evaluated by these instruments, such as daily independence, biographical information, or subjective perception of cognitive impairment, trying to take a holistic view of the patient. For example, BHA includes also BHS, an interview with questions on daily independence, while BHT allows to analyze risk factors for dementia. This aspect of the brain health screening tests appears particularly relevant since brain health is a multidimensional construct, not limited to cognitive dimension. In fact, in projects which aimed at investigating brain health in healthy subjects (The Brain Health Registry [14], The Kaiser Healthy Aging and Diverse Life Experiences - KHANDLE [15], The Brain Health Platform [16], The Barcelona Brain Health Initiative - BBHI [17], the Beijing Aging Brain Rejuvenation Initiative - BABRI [18]and LIFEBRAIN [19]), at least another brain health dimension is evaluated in addition to the cognitive profile. This includes biographical and socio-demographic characteristics, medical, physical or neuroimaging exams performed, risk and resilience factors, lifestyle and healthy measures (mainly quality of life, daily independence, engagement in physical activities, quality of sleep, and nutrition) and mental health (depression, anxiety, and stress) (Table 3).

**Table 3:** Characterization of projects specifically aimed at investigating brain health in healthy subjects focusing on the multiple dimensions considered.

Project	Year of foundation	Country	Population	Characteristics of population	Dimensions investigated
BABRI	2008	CHN	HS	Age: ≥50 Education: ≥6	• Cognitive assessment • Subjective cognitive evaluation
BBHI	2018	ESP	HS	Between 40 and 65 years of age	• Cognitive assessment • Cognitive reserve • Lifestyle and healthy measures • Biographical and socio-demographical information • Mental health measures • Medical and physical exam • Neuroimaging
Brain health platform	2021	USA	HS, MCI, ADRD	Mean age: 74.6 ± 9.5 years Mean education: 15.8 ± 2.6 years	• Cognitive assessment • Cognitive reserve • Medical and physical exam • Biographical and socio-demographical information • Lifestyle and healthy measures
Brain health registry	2014	USA	HS	Age: ≥18	• Cognitive assessment • Daily independence • Lifestyle and healthy measures • Biographical and socio-demographical information • Mental health measures;
KHANDLE	2017	USA	HS	Age: ≥65 Speaking English or Spanish	• Cognitive assessment • Grip strength and gait speed • Cardiovascular risk factors • Engagement in physical activity
LIFEBRAIN	2020	DEU;DNK; ESP; GBR; NLD; NOR; SWE	HS, DEM	Between 0 and 100 years of age	• Neuroimaging • Cognitive profile • Genetics and epigenetics • Lifestyle and healthy measures • Mental health measures

*List of abbreviations:* ADRD, Alzheimer's disease and related disorders; BABRI, Beijing aging brain rejuvenation initiative; BBHI, Barcelona brain health initiative; CHN, China; DEM, Dementia; DEU, Germany; DNK, Denmark; ESP, Spain; GBR, United Kingdom; HS, healthy subjects; KHANDLE, Kaiser healthy aging and diverse life experiences; MCI, mild cognitive impairment; NLD, Netherlands; NOR, Norway; SWE, Sweden; USA, United States of America.

Compared to commonly used cognitive screening, the BHA pays attention to the choice of stimuli that are culturally fair, including ethnically diverse facial stimuli, to reduce cultural bias. The authors of Cogniciti Brain Health Assessment do not explicitly mention the use of culture fair stimuli, but the faces used for the association task belong to different ethnicities. However, further studies are needed to assess the validity of translated and culturally adapted versions in lower educated and culturally diverse populations.

Another strength of the last two instruments mentioned concerns the type of tasks selected.

For example, difficulties in remembering faces, names and preferences of family members or friends are among the most frequent complaints in the elderly. In this sense, compared with the other tools, some tasks could have a better ecological validity as they structure is more similar to the real-life setting such as a face-name association task in Cogniciti Brain Health Assessment or face-food and animals association task in BHA. Indeed, association tests appear more ecological than the learning tests of unstructured stimuli (e.g., a list of words) which are mostly used in the clinical settings. In this way, the assessment may offer a picture of patient's real difficulties in daily life.

Finally, the tablet-based administration modality of BHA and Cogniciti Brain Health Assessment can lead to different advantages. By automating the processes of attribution of scores and of their correction for significant variables, this modality enables faster scoring and reduces the possibility of errors. In addition, the availability of the Cogniciti Brain Health Assessment as a free online tool can make it easy for collecting data on large samples, for example for normative data implementation [13]. It should be kept in mind, however, that the free online availability of the tool may lead to a lower control of data quality. In fact, it is not possible in this way to control the setting in which the test is performed, the person's level of concentration and fatigue, and the degree of comprehension of the task.

Compared with common cognitive screening tests, such as MoCA [20,21] and MMSE [22,23], the current limited diffusion of brain health tools may lead to difficulties in terms of both interpretation of the results (e.g., familiarity with cut-offs and methods used to calculate and visualize the test output, mainly for the computerized tools), and efficacy of the consultation and decision-making processes among different healthcare professionals with various levels of expertise with cognitive tools.

Furthermore, MoCA and MMSE have been translated into many languages and adapted to different cultures [24,25]. Of the screening tests specifically created to investigate brain health, only the BHA is available in more than one language (English, Spanish, and Greek). The reduced dissemination and availability of appropriate translations leads these tests to lack normative data, cut-offs, and validations outside the test's home countries.

## 4 Conclusion

Despite its growing popularity, the concept of brain health is relatively new, the relevant literature is quite sparse, and the various definitions available are often not completely consistent. Further efforts toward a critical revision and homogenization of this concept would be timely and of upmost relevance, and they would clarify the conceptual framework subsiding the development of specific assessment tools.

Interestingly, the WHO brain health definition as a state of brain functioning across multiple dimensions of daily living is in line with the conceptualization of intelligence as a measure of global efficiency of the brain proposed by Royall and colleagues in a series of studies based on structural equation modeling of the associations among intelligence, functional status, and dementia [26]. The possibility that latent factors, such as general intelligence (*g*) and/or the ‘*cognitive correlates of functional status*’ (δ), may underlie the brain health construct might be explored [26]. Given the known role of general intelligence in superintending all cognitive abilities, its relevance on brain health might be of interest, and it may help also refining the brain health conceptual and methodological paradigm.

Indeed, until now, the tests specifically developed for the assessment of the cognitive dimension of brain health are based on tools, methods and procedures that are usually applied to identify cognitive deficits in patients’ populations. On the other side, the brain health construct seems to be more aligned with the idea of enhancing the preservation of an optimal brain integrity and functionality, and thus on the relevance of resources more than deficits.

Further research would be useful both in clarifying the extent to which general latent factors and/or related or independent multidimensional abilities may influence the brain health construct, and in identifying new assessment methods that allow brain health to be considered as a ‘positive’ construct and no longer simply the absence of cognitive impairment.

Despite these criticisms, it is widely recognized that cognitive function is a key dimension of brain health. Several screening tools have been used in the literature to assess the cognitive dimension of brain health which was the focus of this paper. Some of them are well-known and widespread in the neuropsychological field, validated in various clinical setting and translated in many languages.

Others are tools designed and developed ad hoc for the assessment of brain health, with the addition of a more all-inclusive assessment, not limited to cognitive dimensions. They are relatively new, not widely used at present and poorly adapted to different cultures, yet the available validations look promising, as does the fact that their tasks are designed to be ecological and freely available online. Further studies are needed to identify the most appropriate tools for assessing the cognitive dimension of brain health.

## Funding

None.

## Declaration of competing interest

The authors declare that they have no known competing financial interests or personal relationships that could have appeared to influence the work reported in this paper.
